# Brain Stimulation for Emotion Regulation in Adolescents With Psychiatric Disorders: Study Protocol for a Clinical-Transdiagnostical, Randomized, Triple-Blinded and Sham-Controlled Neurotherapeutic Trial

**DOI:** 10.3389/fpsyt.2022.840836

**Published:** 2022-04-25

**Authors:** Lilian Konicar, Karin Prillinger, Manfred Klöbl, Rupert Lanzenberger, Andrea Antal, Paul L. Plener

**Affiliations:** ^1^Department of Child and Adolescent Psychiatry, Medical University of Vienna, Vienna, Austria; ^2^Department of Psychiatry and Psychotherapy, Medical University of Vienna, Vienna, Austria; ^3^Department of Neurology, University Medical Center Göttingen, Göttingen, Germany; ^4^Department of Child and Adolescents Psychiatry and Psychotherapy, Ulm University, Ulm, Germany

**Keywords:** emotion regulation, brain stimulation, transcranial direct current stimulation, HD-tDCS, transdiagnostic, randomized and sham-controlled, triple-blinded neuromodulation

## Abstract

**Background:**

Anxiety, conduct and depressive disorders represent three highly prevalent psychiatric conditions in adolescents. A shared underpinning of these disorders is a shortcoming in emotion regulation, connected to the functioning of the ventromedial prefrontal cortex. Thus, an intervention able to target the suggested neural correlate seems to be highly desirable, aiming to hinder a maladaptive development of emotion regulation abilities and chronification of associated psychiatric disorders. As transcranial direct current stimulation (tDCS) was repeatedly demonstrated as a safe and non-invasive method to modulate specific brain activity, research is in demand to evaluate neurotherapeutic applications in adolescents with psychiatric disorders.

**Method:**

This transdiagnostic, randomized, triple-blind and sham-controlled clinical neurostimulation trial primary aims to investigate if emotion regulation abilities are increased after tDCS in adolescents with psychiatric disorders. Secondly, disorder-specific changes in the anxiety, depression or conduct disorder will be investigated, as well as changes in quality of life, and cognitive and emotional functioning after tDCS intervention. We will include 108 adolescents with psychiatric disorders, displaying a substantial deficit in emotion regulation. Of these, one third each has to be primarily diagnosed with a depressive, anxiety or conduct disorder, respectively. Participants will be randomized to the experimental group (*n* = 54) receiving real anodal tDCS, or to the control group (*n* = 54) receiving sham tDCS. Brain stimulation will be applied for 20 min on five consecutive days twice targeting the ventromedial prefrontal cortex (vmPFC). Changes in emotion regulation, together with changes in disorder-specific clinical symptoms will be recorded by multi-informant psychological ratings. To inspect changes in behavior and gaze, computerized tasks and an eye tracker system will be used. Changes in brain responses to emotional and cognitive stimuli will be examined with three functional magnetic resonance imaging (fMRI) paradigms. In addition, a resting state MRI will be acquired to investigate possible changes in brain connectivity.

**Discussion:**

By investigating “emotion regulation” as transdiagnostic treatment target, this project is oriented toward the Research Domain Criteria framework with a dimensional view on mental illness. The study aims at investigating the potential of tDCS as non-invasive intervention for depressive, anxiety and conduct disorders in adolescents and broadening the scientific foundation for its clinical application.

**Clinical Trial Registration:**

The study is ongoing and has been registered in the German Registry of Clinical Trials (DRKS-ID: DRKS00025601X) on the 28.06.2021.

## Introduction

### Psychiatric Disorders and Emotion Regulation

Given the high prevalence of mental health disorders in children and adolescents with its related psychological strain and suffering, parallel to the still limited efficacy of treatment options, there is a necessity to develop suitable, interventional approaches to support this vulnerable population. Estimates of prevalence of mental health problems before adulthood are generally broad and uneven due to methodological inconsistencies, with values currently ranging from 1 to 51% and a general pooled estimate of around 13.4% (1). In children and adolescents, three groups of mental disorders outstand in their numbers: anxiety (1st), conduct (2nd) and depressive disorders (4th) (1). In Austria, a recent epidemiological study indicated a point prevalence of psychiatric conditions in adolescents of 23.98% (±4.2), where anxiety disorders were most common (9.5%), and depressive (6.1%) and conduct disorders (3.7%) also presented significant amounts (2). The common presentation of anxiety disorders encompasses excessive fear and worrying that hinders global behavioral and social functioning (3), generally afflicting more females than males (4). Depressive disorders are characterized by symptoms such as frequent negative mood, anhedonia and loss of energy, self-deprecating and death thoughts, as well as psychomotor, sleep and appetite disturbances (5), again with a clear female preponderance. Contrary, conduct behavior disorders are more common in male adolescents, displaying diverse forms of externalizing aggression (e.g., fighting, bullying), destructive behavior (e.g., vandalism) and a general violation of rules (6). These three groups of disorders usually result in a heavy public health burden, often remain untreated, and commonly progress further into adulthood (7). Considering this developmental perspective, the importance to intervene as early as possible to hinder a further worsening of the symptoms and the chronification of the disorders into adulthood is obvious.

Conventional treatment approaches branch into two directions: firstly, pharmacological therapies, which are often accompanied by adverse effects, low compliance and off-label-use. Secondly, psychosocial and psychotherapeutical interventions (e.g., cognitive behavioral therapy), which often imply organizational challenges, such as long waiting lists or long treatment durations with related high costs (8).

Converging research in the last years showed that maladaptive emotion regulation is key to development and maintenance of psychopathological conditions, such as depression or anxiety disorder (9, 10). Examples of negative emotion regulation strategies are found across psychopathological states, as rumination in depression, deficit behavioral impulse control in conduct disorders or disengagement bias in anxiety (11). In contrast, successful emotion regulation was shown to act as a significant predictor of academic success/productivity in the classroom [total *R*^2^ = 0.29, *R*^2^ change = 0.07, F_(2, 204)_ change = 20.64, *p* < 0.001, β = 0.27] even after controlling for IQ (12), and was found to be positively related to ambition (*r* = 0.34; *p* < 0.001), to protean career orientation (*r* = 0.26; *p* < 0.001), and to perceived employability (*r* = 0.18; *p* < 0.01) (13), as well as to perceived workplace social support as predictor of job satisfaction and happiness (14).

Psychotherapeutic interventions already demonstrated the feasibility of improving emotion regulation and additionally reducing symptoms of the specific disorder (15). Underlying several psychiatric conditions, emotion dysregulation is a strong candidate for a transdiagnostic construct, and therefore, as a transdiagnostic treatment target (15).

### Neural Basis of Emotion (Dys-) Regulation—The Role of the vmPFC

The central role of the ventromedial prefrontal cortex (vmPFC) in emotion regulation is not only highlighted by its rich connections to cingulate cortex and subcortical structures like the amygdala (16). The vmPFC is shown to regulate the expression of fear responses [e.g., (17)], as well as the volitional suppression of negative emotion [e.g., (18)] by inhibiting amygdala activity i.e., subcortical down-regulation. Furthermore, the vmPFC is involved in critical psychological functions, such as in value-based decision making and social cognition, both highly relevant for modeling behavior depending on emotions (19).

Reduced functioning of the vmPFC, such as in neurological patients with focal vmPFC lesions, is generally linked to diminished emotional responsivity (20), problems to learn about rewards and punishment (21), impaired facial emotion recognition (22), reduced visual attention (23) and to generally reduced social emotions often linked to deficits in empathy, morality and theory of mind (20, 24, 25). In the same vein, aberrant activity in the vmPFC was repeatedly reported in diverse psychiatric disorders, such as in depression and anxiety (26), as well as in conduct disorder in adolescents (27), which further supports the vmPFC as an important common neurophysiological basis for emotion dysregulation in these disorders.

Considering the described domains of psychological (dys)functioning, their role for modeling behavior and their shared neurophysiological basis across the different disorders (19, 28), it is surprising that research regarding suitable neurobiological treatment approaches targeting the vmPFC is still in its infancy.

### Transcranial Direct Current Stimulation and Its Application in Psychiatric Disorders

Transcranial direct current stimulation (tDCS) is a non-invasive brain stimulation technique that uses low intensity currents (~1–4 mA) to reduce or enhance neuronal activity (29) by generating electric fields (30). In tDCS, the current flux is unidirectional and passed by a pair or set of electrodes with a determined electrode polarity, the so-called anodal or cathodal tDCS. This current direction is thought to induce polarity-dependent effects on neuronal excitability, being highly dependent on the network state of the target area (31). Concluding from stimulation of the primary motor cortex and its after-effects, reflected by the amplitude changes of motor evoked potentials, anodal tDCS increases and cathodal tDCS decreases cortical excitability (31). tDCS is also able to alter brain connectivity and thus likely modulate neural communication (32). Studies using resting-state functional magnetic resonance imaging (fMRI) demonstrated the ability of tDCS to alter functional connectivity of the motor and pre-frontal cortex, fronto-parietal and default-mode network (33, 34). Moreover, direct currents were demonstrated to influence ongoing oscillatory activity in several canonical electroencephalographic (EEG) bands such as theta, alpha, beta and gamma (35, 36). Thus, tDCS was reported to lead to acute changes in brain activity, as the neuromodulatory effects begin within seconds after the stimulation starts. On the other hand, physiological aftereffects of tDCS can last for several hours and days (37, 38) and repeated tDCS sessions can even lead to long-term effects via cumulative neuroplastic changes (39).

In the treatment of psychiatric disorders, several brain stimulation techniques are used. More invasive techniques such as electroconvulsive therapy (ECT) are highly effective for severe major depression disorder, but require a sedation of the participant and often have persistent side effects such as cognitive impairment (40). Transcranial magnetic stimulation (TMS), which induces electric current by an electromagnetic field, is less invasive and commonly used in the treatment of depression; side effects are milder but comprise the risk of seizures (40, 41).

tDCS, a stimulation technique known for its only transient and minor side-effects (39), was already tested in several clinical randomized-controlled trials in adult patient with different mental disorders, proving itself as a promising treatment and rehabilitation tool (42). Contrary to basic research investigating the effects of single-session stimulation [e.g., (43)] on general functioning, previous studies using repeated measurements over time showing cumulative neuroplastic benefits of repeated-session tDCS, with mostly 5–10 stimulation session (39).

In depressive disorders, where the left dorsolateral pre-frontal cortex is the most common target for anodal tDCS, a clinical trial showed the superiority of real tDCS over sham stimulation in 60 treatment-resistant patients (44). Several reviews and meta-analyses of randomized clinical trials have now established the safety and acceptability (45), as well as the effectiveness of tDCS compared to sham in alleviating depressive symptoms (42, 46). Most brain stimulation approaches for anxiety disorders used either high or low-frequency repetitive transcranial magnetic stimulation (rTMS) to target the prefrontal cortex (47). Up to date, only one randomized-controlled trial in adults with anxiety disorders was published, where a 5-sessions anodal tDCS treatment targeting the left pre-frontal cortex was not beneficial compared to sham stimulation to reduce anxiety symptoms (48). In a trial including 60 patients with opium use disorder, pre-frontal anodal tDCS combined with methadone was successful in reducing anxiety symptom when compared to sham with methadone and a methadone-only group (49). Up to date, only one tDCS trial in adults with conduct disorders was published and reported reductions in reactive aggression in an aggression task, as well as in self-reported aggression in a forensic population (50).

Encouraging results from studies in adults with psychiatric disorders are driving research into non-invasive brain stimulation (NIBS), particularly TMS and tDCS, in children and adolescents (51). Reviews report feasibility and promising efficacy of these stimulation techniques in various neurologic and psychiatric disorders in children and adolescents such as ASD, ADHD, epilepsy, depression, dyslexia, cerebral palsy and Tourette syndrome (51–53). TDCS proofed to be safe and well-tolerated in this age group. However, up to 2021, no studies with anxiety or conduct disorders were reported in the tDCS adolescent literature.

As a pediatric population might have accelerated neural plasticity after brain stimulation (54), an even greater effect on plasticity after NIBS is expected in children and adolescents. However, regarding the effects and possible side effects on the developing brain, extreme carefulness is warranted when treating this population. Moreover, the immature brain has to be considered as a unique physiological entity, as results from translational studies are missing (52, 55, 56).

Besides the reported literature gap regarding tDCS studies in adolescents with depressive, anxiety or conduct disorders, limitations of previous clinical tDCS studies included missing control groups, insufficient stratification and randomization, the exclusion of female patients, small sample sizes or only unimodal outcome measures (i.e., no psychophysiological or behavioral measures, but solely clinical disorder-specific self-reports) (53, 57, 58).

In line with the framework of the Research Domain Criteria project (RDoC of the United States' National Institute of Mental Health), which orients psychiatric research to uncover the neural systems underlying psychopathology and the behavioral components that flow across diagnostic categories (59), we will investigate the application of tDCS primarily targeting changes in emotion regulation abilities in children and adolescent with psychiatric disorders in general (transdiagnostic approach) as well as separately in each clinical disorder group (i.e., depressive, anxiety and conduct disorder subgroup). As secondary objective, we complementarily aim to investigate possible changes in disorder-specific clinical symptomatology (traditional, nosological approach; i.e., changes in the psychopathology of anxiety, depressive and conduct disorder). Thirdly, we will explore changes in quality of life, as well as in emotional and cognitive functioning in general.

Besides our perquisite for a treatment approach to be safe, non-invasive and free of major side effects, tDCS seems to be also advantageous on a neuroscientific level, because of its neuromodulative character, enabling changes in neural activity in specifically defined target regions. In that sense, a suitable stimulation target for our objectives needs to fulfill two requirements: Firstly, the brain region should play a central role in emotion regulation, integrating emotional and executive functioning (mirrored by strong connections to related brain areas). Secondly, the stimulation target, as a marker for deficient emotion regulation, should subsequently have been reported to be affected in the mentioned clinical populations. Both requirements were fulfilled by one brain region, namely the vmPFC. The vmPFC has not only been shown to be crucial for emotion processing (19), moreover several studies point toward wider regulation capabilities of the vmPFC (60–62), as a center for affective control, integrating emotional and executive functioning (63). Moreover, the vmPFC was reported to be—amongst others—dysfunctional in depression and anxiety (26, 63–65), as well as in conduct disorder in adolescents (27). Based on these theoretical requirements and the first positive investigations using conventional tDCS to target the vmPFC (60, 66–71), the vmPFC arises as an ideal brain stimulation region for the planned intervention which will include an optimized stimulation technique providing even higher focality, namely: HD-TDCS (50, 72).

## Methods and Analysis

The present clinical, interventional trial is designed as a randomized, triple-blind and sham-controlled study. The study protocol has been developed in accordance with the Declaration of Helsinki, approved by the review board of the Medical University of Vienna and registered in the German Registry of Clinical Trials (DRKS-ID: DRKS00025601X).

### Hypotheses

#### Primary Hypothesis

We expect that the participants of the experimental group (in total i.e., transdiagnostic, as well as in each clinical subgroup) will significantly improve in emotion regulation after real tDCS, when compared to the control group (in total i.e., transdiagnostic, as well as in each clinical subgroup) after receiving sham stimulation.

#### Secondary Hypotheses

We expect significant improvements in disorder-specific clinical symptomatology, quality of life, as well as in emotion recognition and regulation, attentional bias and working memory (in the related psychological, behavioral and physiological indices) in the experimental group, compared to the control group after the interventions.

### Participants

A total of 108 participants (*N* = 108) is planned to be recruited primarily from the Department of Child- and Adolescent Psychiatry (Medical University of Vienna, Austria) and private practices via information flyer and verbal study information. Psychological and behavioral pre- post measures, as well as the intervention will be carried out at the same institution. All MRI measures will be conducted at the High-Field MR Centre of the Medical University of Vienna in collaboration with the Department of Psychiatry and Psychotherapy.

#### Sample Size Calculations

Given the planned study design with two treatment conditions (real and sham tDCS), two time points (before and after the intervention) and three diagnostic groups (anxiety, depressive and conduct disorder), a repeated-measures analysis of variance (rmANOVA) with the following parameters was set up: α = 0.0167 (0.05 corrected for three groups), power 1 – β = 0.80, a moderate effect size of f = 0.25 (73). The FEEL-KJ [German version of the Questionnaire for the assessment of emotion regulation in children and adolescents; (74)] as it is used for assessment of the primary endpoint was reported to have a test-retest reliability of r = 0.62, a Chronbach alpha of α = 0.93 for the adaptive strategy subscale and a Chronbach alpha of α = 0.82 for the maladaptive strategies, whereas for the 15 different emotion specific subscales Chronbach alphas between α = 0.69 – α = 0.91 were reported (74). Non-sphericity is of no relevance for only two repeated measures. This, results in a required sample size of 36 per group and 108 subjects overall.

#### Stratification and Randomization

In a first step, participants displaying T-values of the FEEL-KJ Total score outside the clinical norm (T-value <40 or >60 in accordance with the transdiagnostic inclusion criterion of substantial emotion dysregulation) and in addition fulfilling the primary diagnosis of either a depressive, anxiety or conduct disorder, will be included in the study. Secondary diagnosis, especially comorbid disorders related to another subgroup of the current trial (e.g., primary diagnosis: depression and secondary diagnosis: anxiety) will be recorded and taken into consideration for the analysis. Age and sex will serve as stratification variables allocating 12 participants of each clinical group to one age block (age block1: 13 + 14 years; age block 2: 15 + 16 years; age block 3: 17 + 18 years) with half of the participants per age block being female and the other half male.

**Figure 1:**
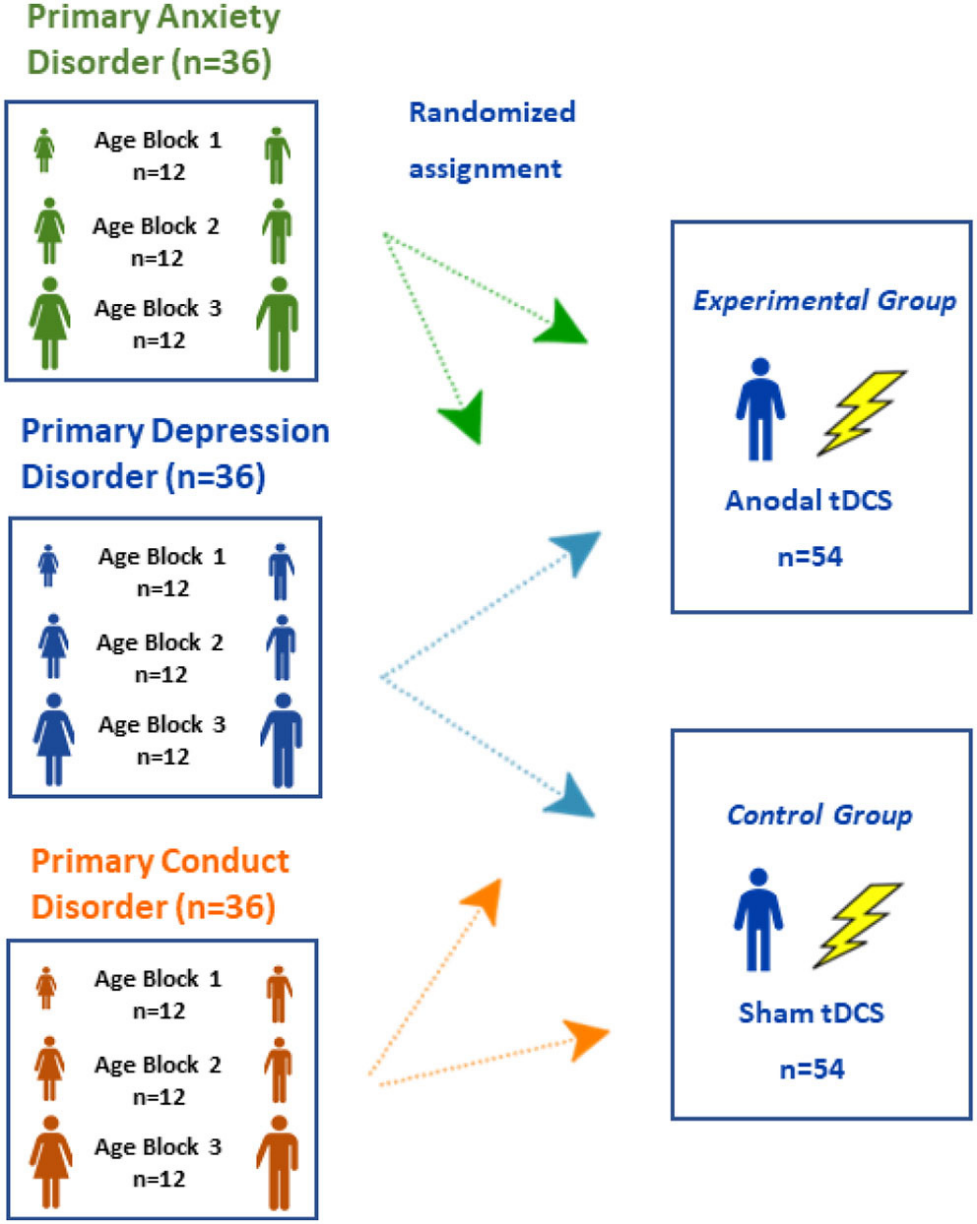
Overview of stratification and randomization of clinical groups.

In a second step, a block randomization is performed: 50% of the participants of each block of each subgroup will be randomly allocated to the real or sham tDCS group, respectively (18 of 36 for each of the three clinical groups, female: male = 1:1). This results in 54 subjects per experimental condition (balanced related to clinical groups, age and sex; see Figure 1).

#### Blinding

For this triple-blind study, the protocols for every participant will be preset using a 4-digit code, which the stimulation device encodes as sham or real stimulation. Neither the participant, nor the study team (investigator, psychologists, physicians) will know which kind of stimulation is applied and will therefore be blind to the type of intervention (sham vs. real). Regarding the analysis of the main hypotheses, also the data analysts will be blinded regarding the experimental group assignment (triple-blind procedure). The blinding and code storage will be done by a staff member not directly involved in the intervention and the day-to-day management of the study. To investigate the success of the blinding, participants and their caregiver and the study team, will be asked about their beliefs of the received stimulation type at the end of the stimulation.

#### Inclusion and Exclusion Criteria

##### Inclusion Criteria

Participants eligible for this clinical intervention study must meet the following criteria^1^:

- male and female adolescents from 13 to 18 years
- with substantial deficits in emotion regulation (T-value <40 or >60 of the FEEL-KJ)
- fulfilling the primary diagnosis of either a depressive disorder (F32, F33), a conduct disorder (F91, F60.2) or an anxiety disorder (F40.1, F41.1) according to the Clinical Description and Diagnostic Guidelines of the ICD 10 (75)
- IQ > 70 (i.e., exclusion of profound, severe, moderate, and mild intellectual disability) on a state-of the art IQ inventory [e.g., WISC/WAIS (76, 77)].
- informed consent and the ability to understand the study information and instructions (sufficient knowledge of German) - signed declarations of consent/assent of study participant and of parent/legal guardian.

##### Exclusion Criteria

- all MRI contraindications such as non-removable metallic devices (cardiac pacemakers, defibrillator, cochlear implant, intracranial/cranial stimulators and other metals, any implant or stainless-steel graft)
- epilepsy or related seizure disorders,
- other severe neurologic diseases or medical conditions (such as chronic migraine, skull defect, craniotomy)
- acute suicidality
- continuous benzodiazepine medication^2^
- concomitant neurofeedback, stimulation intervention or participation in other clinical trials
- failure to comply with the study protocol or to follow the instructions of the investigating team.

### Procedure and Intervention

After study inclusion, stratification and randomization, pre-measures including psychological questionnaires (first visit date), behavioral tests (second visit date) and fMRI paradigms (third visit date) will be conducted. Then, one of the two brain stimulation interventions (real or sham stimulation) will be conducted with each of the participants. In the beginning of every session, the participants will sit down on a chair and complete a brief questionnaire about their general wellbeing, attention, motivation and other life aspects (short mood questionnaires). Afterwards, an electrode stimulation cap will be placed on the head of the participant. Then, the treatment condition is initiated: either real transcranial direct current stimulation (experimental group) or sham (placebo) stimulation (control group). Both brain stimulation interventions will comprise 10 sessions on consecutive days (except weekends), as it was previously done in different successful tDCS treatment trials [summarized in (39)]. After the interventions, the same tasks as for the pre-measurement will be conducted for post-measurements. Follow-up measures, one, respectively, 3 months after the stimulation intervention, will similarly include all tasks as the pre-/ post measurement, except MRI measures.

#### Electrode Placement and Current Strength

Due to the inter-individual variability in the response to tDCS in adult studies (79, 80) as well as the specific features of the stimulation-generated electric fields' behavior in the youth population (81), a suggestion in the pediatric tDCS literature for simulation-based stimulation protocols has been brought up (53, 58). MRI-based finite-element models of the human head can assist parameter choice and dose individualization through the estimation of the spatial distribution and the magnitude of the electric fields (82). In children and adolescents, due to the fast changes in the anatomical characteristics (gray matter alterations, bone thickness, cortical-spinal fluid quantity), field magnitudes are usually 1.5–2 times higher than in the adults (83, 84).

**Figure 2:**
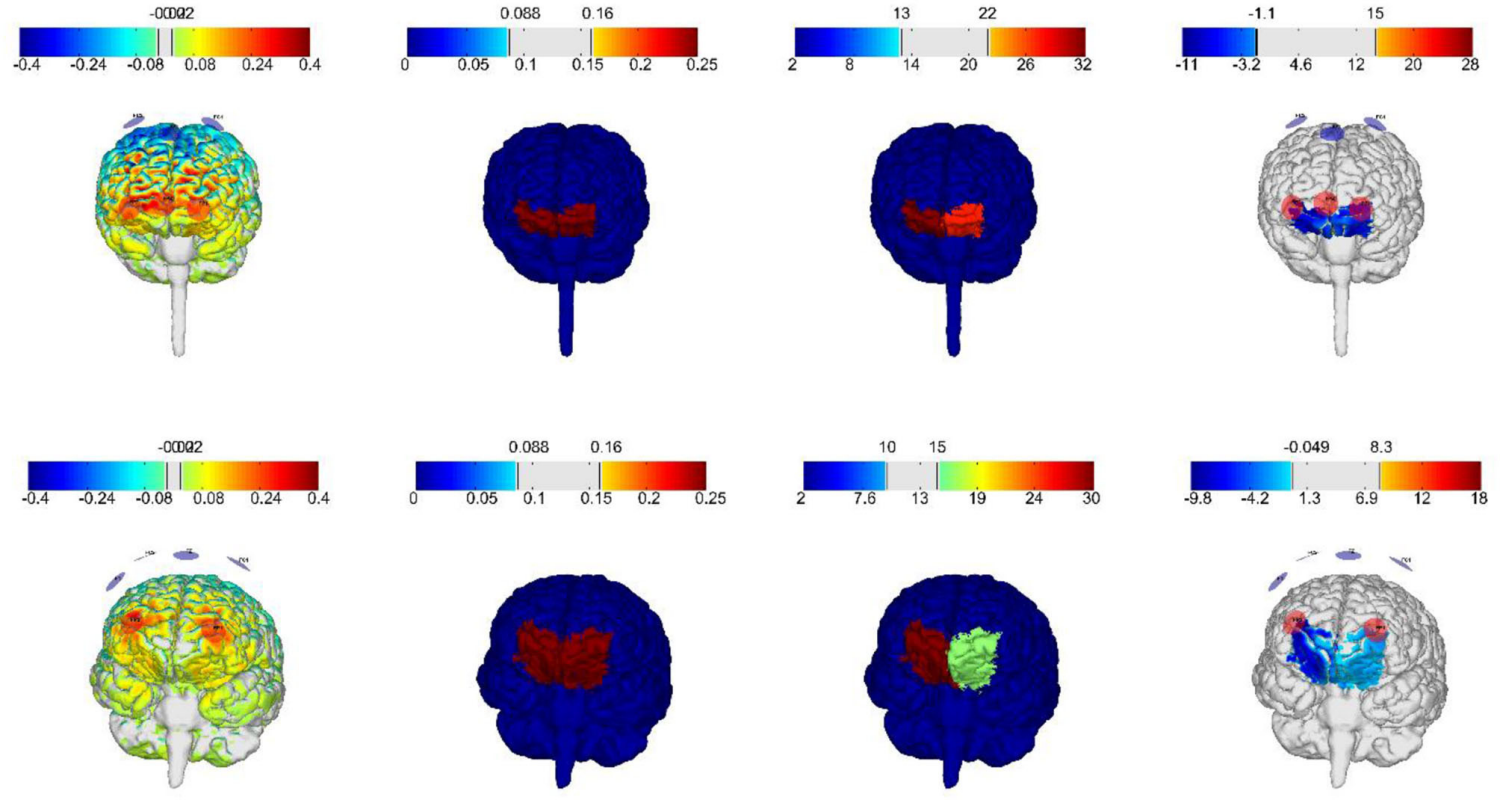
Electric field distributions on gray matter with current intensities within the established safety range for adolescents, generated by the optimized montages for vmPFC from Neuroelectrics. First row: female frontal brain view, second row: male frontal brain view.

Therefore, in this clinical trial, in an attempt to increase spatial precision in targeting the non-superficial cortical area of the vmPFC, an optimized high definition, multi-channel montage based on computational simulations, was chosen. For this reason, the optimization was conducted with the Stimweaver algorithm (Stimweaver montage optimization service from Neuroelectrics NE, Barcelona) to determine the positions of the computed 8 electrodes and required currents for the vmPFC (Figure 2). Based on two head models (male/female) generated from structural T1 images from adolescents within the same age range from a previous clinical trial ran by our research group, an optimized montage for female and male participants was calculated. For female participants anodal stimulation will include electrode positions FP1, FP2, FPz, and P08, while cathodal electrodes will be placed at electrode positions FC1, FC2, and Fz. For male participants anodal stimulation will include electrode positions FP1, FP2, and O1, while cathodal electrodes will be placed at electrode positions FC1, FC2, Fz, and F4. The maximal injected current into the brain at any given time will be below 2.00 mA and maximum current at any electrode is 1.00 mA. The electrodes used in this study are 1 cm radius Ag/AgCl cylindrical electrodes with conductive gel underneath (NG PiStim electrodes).

#### Stimulation Protocol and Intervention Procedure

True anodal tDCS will be applied to the experimental group for 20 min. In the first minute, current will fade-in for 30 s to 2 mA and this current intensity will be maintained. In the 20th minute, tDCS will be reduced and fade-out in the last 30 s. During the sham stimulation in the control group, a fade-in phase for 30 s will be applied, followed by 15 s of 2 mA stimulation and then a fade-out phase for 30 s. Here, participants experience the typical skin sensation during the beginning and thereby will remain unaware of the real condition (85, 86).

The planned 60 min for each session include 20 min of preparation, 20 min of stimulation (real or sham) and 20 min of debriefing. During the 20 min of stimulation, two aspects of practical importance are covered: Firstly, the adolescents should experience the time during brain stimulation as pleasant, appealing and entertaining. Secondly, the longer-lasting effects of tDCS have previously been shown to depend on the emotional and physiological state during tDCS administration (87). Both aspects will be addressed by the following structured intervention procedure: (A) At the beginning of the stimulation, five min of the first part of a child-friendly movie (88) focusing on emotions will be presented to the participants. (B) To further trigger brain areas involved in emotion processing, participants will be asked to assess the emotions of different faces, presented in a naturalistic [videos from EU Emotion Stimuli Set (89) and morphing [clips from FACES Database, (90)] mode (Emotion Recognition Task, ERT). (C) After this expected basic pre-activation of areas involved in general emotion recognition, the complex interplay and link between brain areas involved in emotional functioning (limbic areas, e.g., amygdala) and in parallel brain areas involved in cognitive functioning (e.g., PFC) are aimed to be triggered in parallel by a working memory n-back task with emotional face stimuli (emotional n-back task, e- nBACK). (D) At the end of the stimulation, the next part of the movie will be presented. With this structured intervention procedure, both preconditions for an efficient brain stimulation therapy in adolescents are addressed: the entertaining, as well as the brain-activating function. Further, the randomized and adaptive character of the applied stimuli will, together with the presentation of the full movie (divided into 10 sessions), facilitate focused engagement of the participants during the repeated stimulation sessions and standardize the activity during the stimulation across all participants. Additionally, study motivation in form of sugar-free candies, small give-aways and vouchers from cooperating institutions will be given to the participants at the end of each session.

#### Safety and Adverse Events

When safety guidelines are followed, tDCS is considered a safe stimulation modality for children and adolescents as well as for adult patients with various neurological conditions and healthy subjects, as studies prove in over 18,000 tDCS sessions in total (91). In addition, the lack of reported serious adverse events, also feasibility, tolerability and safety of tDCS in children and adolescents were positively evaluated (53, 58, 92). The mild and transient, reported adverse effects of tDCS in children and adolescents included tingling (11.5%), itching (5.8%), redness (4.7%) and scalp discomfort (3.1%) were similar to those in adults (93). For the reporting of any possible adverse effect, the German version of the questionnaire of sensations related to transcranial electrical stimulation is used (accessible at: http://neurologie.uni-goettingen.de/downloads.html). Unlikely events such as serious adverse events or adverse reaction related to the device will be reported to the Ethics Committee of the Medical University of Vienna, as well as to the Austrian Agency for Health and Food Safety (AGES). Nevertheless, as tDCS is a non-invasive method and free from major side effects, no serious health risks for the adolescent study participants of this study are expected. In the same vein, MRI measures are non-invasive and safe as long as the necessary precautions are followed; bearing no expected health risks for the examined study participants.

### Instruments

To complement the determined, unimodal measure for the investigation of the primary hypotheses of this study, our study exceeds the typical outcome measures of tDCS studies (i.e., self-report-questionnaires) with a mix of methods on multiple levels will be applied before and after the therapeutic intervention for the remaining objectives of this study. Psychological self- and parental ratings will detect changes in emotion regulation, empathy, psychiatric symptoms and quality of life (multi-informant). Changes in behavioral reactions and gaze behavior will be measured with five computerized, psychological tasks. Neurophysiological changes in brain responses to emotional and cognitive stimuli will be examined with three fMRI paradigms. In addition, a resting-state MRI before and after the brain stimulation intervention will be used to gain insight into potential changes in brain functional connectivity.

#### Psychological Self- and Parental Ratings

To provide an integral view on the ability of emotion regulation, multi-informant assessment via children and parental ratings will be compiled. Further we aim to not only investigate the ability to regulate emotions and related emotion regulation strategies [via FEEL KJ / P: (74, 94), ERQ (95), ERC (96), ASQ-Y (97)] but also to detect difficulties in emotion regulation [via DERS (98)] and combine those indices to a holistic view on the individual's emotion regulation abilities. The FEEL questionnaire was selected as the primary outcome, as it provides not only a scale for adaptive and maladaptive strategies for emotion regulation, but more importantly, it provides the emotion regulation scores separately for three emotions, namely: anger, anxiety and sadness—mirroring the three- targeted clinical subgroups (conduct disorder, anxiety disorder and depressive disorders).

Multi-informant ratings and interviews will be also used regarding general psychopathology [YSR/CBCL, (99); Interview: KINDER DIPS (100)] and disorder-related symptoms and comorbidities, such as anxiety, depression, impulsivity and aggression [DISYPS III—SSV, DES, ANG, self- and parental rating versions (101), BDI (102), STAIK / STAI (103, 104), STAXI-2KJ / STAXI II (105, 106)].

We will also track possible changes in different aspects of empathy as a general supportive factor for emotion regulation [via IECA (107), ICU-C/P (108, 109), BES (110), GEM (111)], as well as quality of life as a proxy for treatment impact (96), with both self-report and parental questionnaires [KIDSCREEN (112)].

#### Eye Tracking and Behavioral Measures

##### Frankfurter Test and Training for Recognizing Facial Affect II

In this computerized task, the subjects have to identify basic emotions presented on 50 black and white photos of the entire face (113). The used stimuli are based on Ekman's concept basic emotions [happiness, sadness, anger, disgust, fear, surprise and neutral; (114)] and the test includes measurements of reaction times and correct classification.

##### Emotional Stroop Task

Here we adapt an emotional conflict task (implicit regulation), as prior used to detect the activation of medial pre-frontal regions during emotion regulation (115, 116). This paradigm is built as a variation of the STROOP task. Participants have to identify happy or fearful faces, and are instructed to ignore the overlaid words “fear” and “happy” (“ANGST” or “GLÜCK” in German) *via* button pressing. A trial-by-trial adaptation to emotional conflict brings up an emotional regulatory mechanism (116). Here, behavioral reactions (correct/incorrect classification, omission) will be recorded.

##### Emotional n-back Task

Cognitive and emotional capacities will be investigated using a modified version of a working memory n-back task (117) with emotional face stimuli pre and post brain stimulation intervention. This paradigm was developed as a modified emotional n-back task, which adopts to the current performance of the patient, varying the degree of difficulty adaptively during each stimulation session. During the task, behavioral reactions such as button presses (correct/ incorrect classification, omission) and reaction times will be recorded.

##### Attentional Bias Task

To investigate attentional bias, a static faces free-viewing task will be applied, where participants are instructed to observe the simultaneous presentation of a neutral facial expression and an emotional facial expression (angry, sad or happy faces). Changes in the direction of initial gaze, total fixation duration (118) and pupil dilation will be measured via eye tracking system (Tobii Group, Danderyd, Schweden).

##### Emotion Recognition Task

Emotions of different static and morphing faces displaying different emotional states will be presented to the participants to investigate the perception of emotions in others. The emotional stimuli will be taken from validated Databases [EU Emotion Stimuli Set (89); FACES Database (90)], and will be presented at pre and post measures. Besides behavioral reactions (correct/ incorrect classification, omission, reaction times), also gaze behavior, fixation duration and pupil dilation will be recorded via eye tracking system (Tobii Group, Danderyd, Schweden).

#### fMRI Procedure and Measures

MRI data will be acquired via a 3T Siemens MAGNETOM Prisma MR Scanner (Siemens, Erlangen, Germany) equipped with a 64-channel head coil. Sequence optimization will be conducted in accordance with the recommendations given by the Human Connectome Project (119) to allow for highly improved temporal and spatial resolution, leading to overall increased sensitivity and specificity for the subsequent analyses.

The subjects will first be explained the tasks and have trial runs outside of the scanning room. After a briefing on the correct behavior inside the gantry and a safety check, subjects will be transferred onto the scanner bed and placed in the MRI scanner. For task responses, subjects are given an MR-compatible button box (Current Designs Inc., 3950 Haverford Ave. Philadelphia) which rests on their belly while in the scanner. The experimenters keep visual contact during the examinations and track the responses on the stimulus laptop. Stimuli are presented via a beamer and mirror system on a screen placed at the rear end of the gentry.

The MRI scans are planned in the following order: The e-nBACK is conducted first as it is expected to be the most cognitive demanding task followed by the eSTROOP as the second most demanding one. To allow for a break, the T1-weighted image is acquired next. Afterwards, the RS data is recorded, where the break during the T1-weighted image should also mitigate carryover effects from the previous task. The ERT isacquired last.

The fMRI implementation of the e-nBACK task will only consist of a 2-back and an additional 0-back condition as control organized in a mixed design [similar to (120)]. The conditions will be presented in eight blocks (four per condition) of 12 stimuli, where the pictures are shown for 2 s with 0.5 s between. Altogether, 96 stimuli will be shown. Blocks will be separated by baseline periods of 30 s. The whole task takes 8.5 min.

The fMRI adaptation of the eSTROOP task is taken from Alder et al. (121) respectively, Almdahl et al. (120): In short, 149 stimuli will be presented for 1 s each with jittered baseline periods of 3–5 s in an event-related manner. With additional baselines of 10 s at the beginning at end, the task will take ~13 min.

For the resting-state scan, subject will be told to lie as still as possible and let their mind wander. They are additionally shown the “Inscapes” video which was validated for fMRI acquisition and shown to significantly reduce in-scanner head movement (122). The acquisition takes 7 min.

In the fMRI version of the ERT, only the morphing faces will be used. A total of 42 stimuli will be presented in an event-related design, where the pictures are shown for a maximum of 2 s but should be interrupted via button press as soon as the subjects have identified the target emotion. After the stimulus and a short baseline of 1 s, the subjects have to select the target emotion out of six options. Stimulus-response pairs are interleaved with a jittered baseline of 5–10 s. The duration of the task strongly depends on the response times of the subjects but was ~10 min in previous investigations (123).

### Outcomes

The primary outcome of this study is the change in emotion regulation ability, measured by the raw score of the FEEL-KJ (74). Here, we expect an increase in emotion regulation after the intervention of the experimental group in total (as well as for each clinical experimental subgroup) compared to the control group in total (as well as for each clinical control subgroup).

As exploratory outcomes, we will investigate if the application of tDCS improves the disorder-related clinical symptomatology, quality of life or emotion regulation, whereas changes in raw scores of the related self- and parental reports (as summarized in Psychological Self- and Parental Ratings) are defined as secondary endpoints.

Further we will explore if indices of emotion recognition [measured via Frankfurter Test and Training for Recognizing Facial Affect (FEFA II) and the Emotion Recognition Task (ERT)], and (implicit) emotion regulation (measured via eSTROOP) improve from before to after tDCS stimulation. In addition, measures of attentional bias [*via* Attentional Bias Task (ABT)] and working memory (*via* e n-BACK) will give insight regarding potential improvements in the related indices from before to after tDCS stimulation. Indices of each task (such as the number of correct behavioral assignments, commission, omission, reaction times, eye gaze fixation duration, pupil dilation, brain activation, brain connectivity) are summarized in Eye Tracking and Behavioral Measures and fMRI Procedure and Measures.

### Statistical Analysis

Regarding the primary endpoint, repeated-measures analysis of variance (rmANOVA) with Time (pre/post) and Treatment Condition (real stimulation/sham stimulation), as well as considering the three clinical subgroups will be conducted to investigate the interaction between times and groups on the dependent variable [FEEL-KJ raw scores (74)]. The sex and age of the participants will be used as blocking factor accounting for treatment variability between males and females and the three age groups, respectively. In the case of distributions not suitable for conventional testing and missing values, a generalized linear mixed model on the relationship between the stimulation condition and behavioral variables can be applied. Stimulation condition and time will be taken as the fixed-effect factors and the participant's ID as the random-effect factor. Parameters of the model will be fitted with the maximum-pseudolikelihood method, the appropriate distribution and link function according to the characteristics of the response data. A marginal ANOVA will follow to evaluate the fix-effect factors significance. The secondary endpoints, i.e., the indicators for aggression, depression, anxiety and quality of life, as well as cognitive and emotional functioning will be compared using the same approach. In order to identify the direction of effects, *post-hoc* tests will be employed.

Single-subject analysis of the fMRI data will be conducted using the general linear model (GLM) as implemented in SPM12. The conditions will be modeled as previously described for the eSTROOP (120), e-nBACK (124) and the ERT [similar to (123)]. Image-derived movement estimates and surrogates of physiological artifacts (heartbeat, breathing) will be used as covariates and regressed out. The same corrections will be applied to the RS data, which will be used for estimating functional connectivity using in-house software. Group analyses of the whole-brain fMRI data will be again conducted using the GLM in SPM12. The Sandwich Estimator (SwE) will additionally be utilized for missing data. Diagnostic group and measurement (pre-/post-intervention) will be used as model factors. Subject age and sex will further be corrected for on group-level. Besides factorial group analysis, exploratory correlations will be calculated with indicators of disorder-specific impairments and emotion regulation capabilities. For multiplicity correction, SPM12 uses random field theory based on the smoothness of the data and SwE a bootstrapping approach.

## Discussion

Here, a transdiagnostic, randomized, triple-blinded and sham-controlled neurotherapeutical brain stimulation trial is presented. From a translational perspective, this study promises high rewards to both: basic research in neuroscience as well as the fields of mental health. Regarding the former, by targeting one of its assumed neural substrates, we aim to add causal evidence to the involvement of the vmPFC in pathologies of emotion regulation. Further, we would establish a proof that anodal tDCS in combination with an optimized, multi-electrode montage can efficiently affect the targeted emotion regulation deficits via the modulation of one of its neural underpinning, namely the vmPFC. In contrast to previous tDCS studies, the simulation and modeling for the multi-electrode montages were based on MRI measures of both sexes, as well as in the optimal age range, adding specificity to the stimulation protocol. Electrical stimulation protocols aiming to target neural networks for e.g., changing the inhibitory/ excitatory balance in different brain regions [e.g., (125)] would be interesting for future endeavors, if evidence is repeatedly shown.

As the intervention has promising applications in psychiatry, it is important to point out, that the current project provides the first empirical, transdiagnostic tDCS therapy attempt in adolescents with depressive, anxiety and conduct disorders. With this approach, we aim to overcome disadvantages of standard therapies like side effects of or insufficient commitment to medication, long durations and related costs. Therefore, we developed a specific therapeutic brain stimulation procedure to achieve the greatest possible commitment on the patients' side, as well as the greatest possible neuromodulative effect. The training paradigm is built for the purpose to activate cognitive and emotional brain networks at the same time: brain regions involved in cognitive functioning (e.g., prefrontal areas), through the required actions during the tasks and at the same time brain regions involved in emotional functioning (limbic areas, e.g., amygdala), through the presentation of emotional stimuli. With this procedure, we expect to activate those brain areas through the training paradigm, which are simultaneously stimulated with tDCS.

Here, specific training effects have to be considered regarding potential biases in outcome measures. Both treatment groups undergo the described emotion regulation training, regardless of receiving real or sham stimulation. Therefore, we will not be able to reduce our results to the pure effect of the activation via tDCS since an induced modulatory training effect is actually desired. Moreover, the emotion regulation training itself might lead to improvements in both groups. If group differences in the improvements of the clinical outcome measures become insignificant, future research will be needed to differentiate between the training and stimulation influences. Only after this analysis, it is possible to clarify if only the developed emotion-cognition training procedure alone is sufficient for an effective training or if the brain stimulation further increases the therapeutic effect. In this case, also the influence of the engagement in the study *per se* would need to be investigated via a suitable control condition.

As the here presented clinical therapy trial not only includes three groups of patients and a novel brain stimulation procedure, but also pre- and post-measures on multiple levels (subjective psychological measures via self- and parent-rated questionnaires, behavioral and eye tracking tests, neuronal measures via MRI), inclusions of further diverging control groups would have gone beyond the scope of this single study.

Considering the developmental psychophysiological perspective, this project does not only highlight the treatment advantages of the excitability of the adolescent brain, it also takes the chance to possibly intervene early enough to hinder a further worsening of the clinical symptoms of depressive, anxiety or conduct disorders and the pathological manifestations in adulthood.

Utilizing a transdiagnostic approach for the treatment of emotion dysregulation as a common factor in anxiety, depressive and conduct disorders, this study is oriented toward a dimensional perspective on mental health as it was formalized in the Research Domain Criteria framework. If successful, this project will provide a non-invasive therapeutic option for the most prevalent psychiatric disorders in adolescents, with the possibility to transfer the protocol to related conditions in future studies.

## Trial Status

The described study started in November 2021; start of recruitment is planned for the beginning of 2022.

## Ethics Statement

The study was approved by the Ethics Committee of the Medical University of Vienna and the Austrian Agency for Health and Food Safety (AGES) and will be conducted according to the Declaration of Helsinki.

## Author Contributions

LK and KP designed the trial. KP, MK, AA, and LK developed, programmed, piloted the therapeutic training paradigm, and defined the stimulation parameters. Neuroelectrics simulated and developed the stimulation model accordingly. LK and KP coordinate the recruitment of the participants. LK, KP, and MK are responsible for data collection and the planning of the data analyses. LK, RL, MK, and KP will coordinate the MRI examinations. LK, KP, MK, AA, RL, and PP interpret the data, wrote, edited, and revised the paper. All authors approved the final manuscript.

## Funding

This project is supported by the Austrian Science Fund (FWF): KLI960B.

## Conflict of Interest

The authors declare that the research was conducted in the absence of any commercial or financial relationships that could be construed as a potential conflict of interest.

## Publisher's Note

All claims expressed in this article are solely those of the authors and do not necessarily represent those of their affiliated organizations, or those of the publisher, the editors and the reviewers. Any product that may be evaluated in this article, or claim that may be made by its manufacturer, is not guaranteed or endorsed by the publisher.

## Footnotes

^1^As right-handed and left-handed participants will be included in this study, handiness will be measured via the Edinburgh inventory (78) for potential further analysis.

^2^Additional medication information: (-) Allowed: SSRIs if dose is stable for at least 4 weeks prior to study participation. Antipsychotics as pro-re-nata medication received occasionally (not in the week before the first stimulation and only if they did not receive 500 mg chlorpromazine equivalent in the month before the first stimulation) or continuous antipsychotic medication, if dose is stable for at least 4 weeks prior to study participation. Methylphenidate, any amphetamine preparation and melatonin are allowed, if dosage is stable for at least 1 week prior entering the study. (-) Dosage changes throughout the study will lead to exclusion.
