# Supplementary material for: Brain Stimulation for Emotion Regulation in Adolescents With Psychiatric Disorders: Study Protocol for a Clinical-Transdiagnostical, Randomized, Triple-Blinded and Sham-Controlled Neurotherapeutic Trial
Source: Front Psychiatry. 2022 Apr 25;13:840836. doi: 10.3389/fpsyt.2022.840836 (PMC9082670; doi:10.3389/fpsyt.2022.840836)
Supplement: Supplementary file 1 [file Table_1.pdf]

SUPPLEMENRATY MATERIAL

Table 1. Psychological Multi-informant Assessment: Self-Reports (“S”) and Parental Reports (“P”)

Abbreviation	Questionnaire Name (S= Self report / P= Parental Report)	Psychological Index Targeted Psy. Construct	Psychometric Property
**FEEL-KJ**	Fragebogen zur Erhebung der Emotionsregulation bei Kindern und Jugendlichen (S+P)	Emotion Regulation	**(-) Cronbachs Alpha[1]:**<br>-) Different emotion specific subscales: α = .69 - =.91<br>-) Adaptive strategy subscale: α = .93<br>-) Maladaptive strategy subscale:α = .82<br><br>**(-) Test-Retest Reliability**<br>-) Different emotion specific subscales: r_{tt} = .62 - r_{tt} = .81<br>-) Adaptive strategy subscale: r_{tt} = .81<br>-) Maladaptive strategy subscale: r_{tt} = .73<br>*(Grob & Smolenski, 2009)*
**ERQ**	Emotion Regulation Questionnaire (S+P)	Emotion Regulation	**(-) Cronbachs Alpha[1]:** α = .74-.76<br>*(Abler & Kessler, 2009)*
**ERC**	Emotion Regulation Questionnaire (P)	Emotion Regulation	**(-) Cronbachs Alpha[1]:**<br>-) N/L scale: α = .96<br>-) ER scale: α = .83<br>-) Overall α = .89<br>*(Reis et al., 2016)*
**ASQ-Y**	Affective Style Questionnaire (S)	Emotion Regulation	**(-) Cronbachs Alpha[1]:**<br>Subscale Suppression: α = .77<br>Subscale Adjustment/Reappraisal: α = .76<br>Subscale Acceptance: α = .76<br>*(Graser et al., 2019)*
**DERS**	Difficulties in Emotion Regulation Scale (S)	Diff. In Emotion Regulation	**(-) Cronbachs Alpha[1]:** α = .0.93<br>**(-) Test-Retest Reliability** r_{tt} = .88<br>*(Gratz & Roemer, 2004)*
**YSR / CBCL**	Youth Self Report / Child-Behavior Checklist (S+P)	General Psychopathology	**YSR**<br>**(-) Internal Consistency:**<br>-) “internalizing behavior” + “externalizing behavior” r=.86<br>-) "Aggressive Behavior" + "Anxiety/Depression" + "Physical Ailments" + "Antisocial Behavior" + "Attention problems" r>.70

			(*YSR - Fragebogen Für Jugendliche – Hogrefe Verlag*, n.d.)<br>**CBCL**<br>**(-) Internal Consistency:** r >.85<br>*(Döpfner et al., 2014)*
**DISYPS III: ANZ**	Diagnostic system for mental disorders according to ICD-10 and DSM-IV for children and adolescents – III (S+P)	Anxiety	**(-) Cronbachs Alpha[1]:**<br>All subscales between: α = .70 -. α = .90<br>*(Döpfner & Görtz-Dorten, 2017)*
**DISYPS III: DES**	Diagnostic system for mental disorders according to ICD-10 and DSM-IV for children and adolescents – III (S+P)	Depression	**(-) Cronbachs Alpha[1]:**<br>All subscales between: α = .70 -. α = .90<br>*(Döpfner & Görtz-Dorten, 2017)*
**DISYPS III: SSV**	Diagnostic system for mental disorders according to ICD-10 and DSM-IV for children and adolescents – III (S+P)	Conduct Disorder	**(-) Cronbachs Alpha[1]:**<br>All subscales between: α = .70 -. α = .90<br>*(Döpfner & Görtz-Dorten, 2017)*
**STAIC / STAI**	STAIC: State-Trait Anxiety Inventory for Children (6-14 yrs) (S)<br>STAI: State-Trait Anxiety (>15 yrs) (S)	Anxiety	**STAIC:**<br>**(-) Cronbachs Alpha[1]:** α = .0.81<br>**(-) Test-Retest Reliability** r_{tt} = .64<br><br>**STAI:**<br>**(-) Cronbachs Alpha[1]:** α = .90<br>**(-) Test-Retest Reliability**<br>r_{tt} =0.77–0.90 („Trait"),<br>r_{tt} =0.22–0.53 („State")<br>*("Mittenzwei, K., 2013)* | *Ediss.Sub.Hamburg, n.d.)*
**DIKJ / BDI**	DIKJ: Depression inventory for children and adolescents (8-16 yrs) (S)<br>BDI: Beck Depression Inventory II (>13yrs) (S)	Depression	**(-) Cronbachs Alpha[1]:**<br>-) Depressive Patients: α = .93 (*N* = 288)<br>-) Patients other primary psy. Diagnosis: α = .92 (*N* = 123)<br>-) Healthy Subjects: α = . 90 (*N* = 582)<br>**(-) Test-Retest Reliability** r_{tt} = .78<br>(*BDI-II - Beck-Depressions-Inventar Revision – Hogrefe Verlag*, n.d.)
**STAXI-2 KJ / STAXI-2**	STAXI-2-KJ: State-Trait-Anger Inventory for Children and Adolescents (9-16 yrs)(S)<br>STAXI-2: State-Trait-Anger Inventory (<16yrs) (S)	Aggression - Anger	**(-) Cronbachs Alpha[1]:** α = .71-.88<br>**(-) Test-Retest Reliability** r_{tt} = .33-.53<br>(*STAXI-2 - Das State-Trait-Ärgerausdrucks-Inventar - 2 – Hogrefe Verlag*, n.d.; *STAXI-2 KJ - Das State-Trait-Ärgerausdrucks-Inventar - 2 Für Kinder Und Jugendliche – Hogrefe Verlag*, n.d.)
**ICU-Y / ICU-P**	Inventory of Callous-unemotional traits (S+P)	Aggression-Callus Unemotional Traits	**ICU-Y**<br>**(-) Cronbachs Alpha[1]:**<br>Total: α = .83<br>Subscale Uncaring α = .80<br>Subscale Callous α = .75,<br>Subscale Unemotional α = .71<br><br>**ICU-P**<br>**(-) Cronbachs Alpha[1]:**<br>Total: α = .80<br>Subscale Uncaring α = .80<br>Subscale Callous α = .75,

			Subscale Unemotional α = .65<br>*(Cardinale & Marsh, 2020)*
**IECA / GEM-PR**	Bryant Empathy Index for Children and Adolescents (S)<br>Griffith Empathy Measure - Parent Report (P)	Empathy	**IEAC**<br>**(-) Cronbachs Alpha[1]:**<br>Subscale Understanding of feelings: α = .76<br>Subscale Feelings of sadness: α = .83<br>Subscale Tearful reaction: α = .78<br>**(-) Test-Retest Reliability**: r_{tt} = .79<br><br>**GEM**<br>**(-) Cronbachs Alpha[1]:** α = .81<br>**-)** Subscale Cognitive Empathy α = .62<br>-) Subscale Affective Empathy α = .83<br>(*Lucas-Molina et al., 2016; Schwenck et al., 2011)*
**BES**	Basic Empathy Scale (S)	Empathy	**(-) Cronbachs Alpha[1]:**<br>-) Cognitive Scale: α = .79<br>-) Affective Scale: α = .85<br>*(Jolliffe & Farrington, 2006)*
**KIDSCREEN-52**	Quality-of-life measure for children and adolescents (S+P)	Quality of Life	**(-) Cronbachs Alpha[1]:** α = .77 - α = .89<br>**(-) Test-Retest Reliability** rtt = .56 - .77<br>**(-) Scaling success (MAP):** >97.8% for all dimensions<br>**(-) Rasch analysis item fit (INFITmsq):** 0.80 - 1.27<br>**(-) Intraclass correlation coefficients:** 0.56 - 0.77<br>*(Ravens-Sieberer et al., 2008)*

References

Abler, B., & Kessler, H. (2009). Emotion Regulation Questionnaire – Eine deutschsprachige Fassung des ERQ von Gross und John. *Diagnostica*, *55*(3), 144–152. https://doi.org/10.1026/0012-1924.55.3.144

BDI-II - Beck-Depressions-Inventar Revision – Hogrefe Verlag. (n.d.). Retrieved February 10, 2022, from https://www.testzentrale.de/shop/beck-depressions-inventar.html

Cardinale, E. M., & Marsh, A. A. (2020). The Reliability and Validity of the Inventory of Callous Unemotional Traits: A Meta-Analytic Review. *Assessment*, *27*(1), 57–71. https://doi.org/10.1177/1073191117747392

Döpfner, M., & Görtz-Dorten, A. (2017). *DISYPS-III – Diagnostik-System für Psychische Störungen nach ICD-10 und DSM-5 für Kinder und Jugendliche – III*. Hogrefe.

Döpfner, M., Görtz-Dorten, A., & Lehmkuhl, G. (2008). *DISYPS-II Diagnostik-System für psychische Störungen nach ICD-10 und DSM-IV für Kinder und Jugendliche - II*. Huber.

Döpfner, M., Plück, J., & Kinnen, C. (2014). *Elternfragebogen über das Verhalten von Kindern und Jugendlichen (CBCL/6-18R), Lehrerfragebogen über das Verhalten von Kindern und Jugendlichen (TRF/6-18R), Fragebogen für Jugendliche (YSR/11-18R)*. Huber.

Graser, J., Heimlich, C., Kelava, A., Hofmann, S. G., Stangier, U., & Schreiber, F. (2019). Erfassung der Emotionsregulation bei Jugendlichen anhand des „Affective Style Questionnaire – Youth (ASQ-Y)“. *Diagnostica*, *65*(1), 49–59. https://doi.org/10.1026/0012-1924/a000210

Gratz, K. L., & Roemer, L. (2004). Multidimensional Assessment of Emotion Regulation and Dysregulation: Development, Factor Structure, and Initial Validation of the Difficulties in Emotion Regulation Scale. *Journal of Psychopathology and Behavioral Assessment*, *26*(1), 41–54. https://doi.org/10.1023/B:JOBA.0000007455.08539.94

Grob, A., & Smolenski, C. (2009). *FEEL-KJ: Fragebogen zur Erhebung der Emotionsregulation bei Kindern und Jugendlichen*.

Jolliffe, D., & Farrington, D. P. (2006). Development and validation of the Basic Empathy Scale. *Journal of Adolescence*, *29*(4), 589–611. https://doi.org/10.1016/J.ADOLESCENCE.2005.08.010

Lucas-Molina, B., Pérez-Albéniz, A., Giménez-Dasí, M., & Martín-Seoane, G. (2016). Bryant’s Empathy Index: Structure and Measurement Invariance across Gender in a Sample of Primary School-Aged Children. *The Spanish Journal of Psychology*, *19*. https://doi.org/10.1017/SJP.2016.44

Ravens-Sieberer, U., Gosch, A., Rajmil, L., Erhart, M., Bruil, J., Power, M., Duer, W., Auquier, P., Cloetta, B., Czemy, L., Mazur, J., Czimbalmos, A., Tountas, Y., Hagquist, C., Kilroe, J., Fuerth, K., Simeoni, M. C., Robitail, S., Nickel, J., … Phillips, K. (2008). The KIDSCREEN-52 Quality of Life Measure for Children and Adolescents: Psychometric Results from a Cross-Cultural Survey in 13 European Countries. *Value in Health*, *11*(4), 645–658. https://doi.org/10.1111/J.1524-4733.2007.00291.X

Reis, A. H., De Oliveira, S. E. S., Bandeira, D. R., Andrade, N. C., Abreu, N., & Sperb, T. M. (2016). Emotion Regulation Checklist (ERC): Preliminary Studies of Cross-Cultural Adaptation and Validation for Use in Brazil. *Temas Em Psicologia*, *24*(1), 97–116. https://doi.org/10.9788/TP2016.1-07

Schwenck, C., Schmitt, D., Sievers, S., Romanos, M., Warnke, A., & Schneider, W. (2011). Kognitive und emotionale Empathie bei Kindern und Jugendlichen mit ADHS und Störung des Sozialverhaltens. *Http://Dx.Doi.Org/10.1024/1422-4917/A000118*, *39*(4), 265–276. https://doi.org/10.1024/1422-4917/A000118

STAXI-2 - Das State-Trait-Ärgerausdrucks-Inventar - 2 – Hogrefe Verlag. (n.d.). Retrieved August 19, 2021, from https://www.testzentrale.de/shop/das-state-trait-aergerausdrucks-inventar-2.html

STAXI-2 KJ - Das State-Trait-Ärgerausdrucks-Inventar - 2 für Kinder und Jugendliche – Hogrefe Verlag. (n.d.). Retrieved August 19, 2021, from https://www.testzentrale.de/shop/das-state-trait-aergerausdrucks-inventar-2-fuer-kinder-und-jugendliche-76359.html

“The Screen for Child Anxiety Related Emotional Disorders” (SCARED) : eine deutsche Übersetzung und erste empirische Anwendung des Fragebogens für Angststörungen im Kindes- und Jugendalter (SCARED-D) | ediss.sub.hamburg. (n.d.). Retrieved February 10, 2022, from https://ediss.sub.uni-hamburg.de/handle/ediss/5180

YSR - Fragebogen für Jugendliche – Hogrefe Verlag. (n.d.). Retrieved February 10, 2022, from https://www.testzentrale.de/shop/fragebogen-fuer-jugendliche.html
